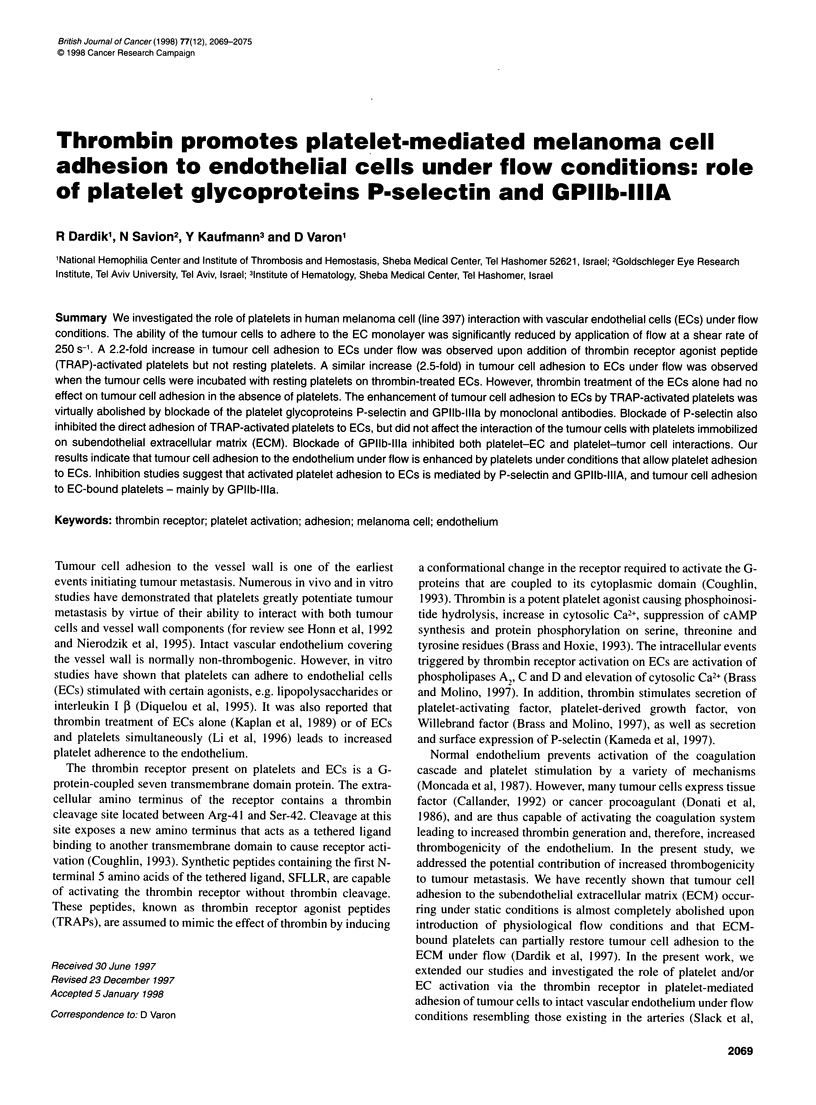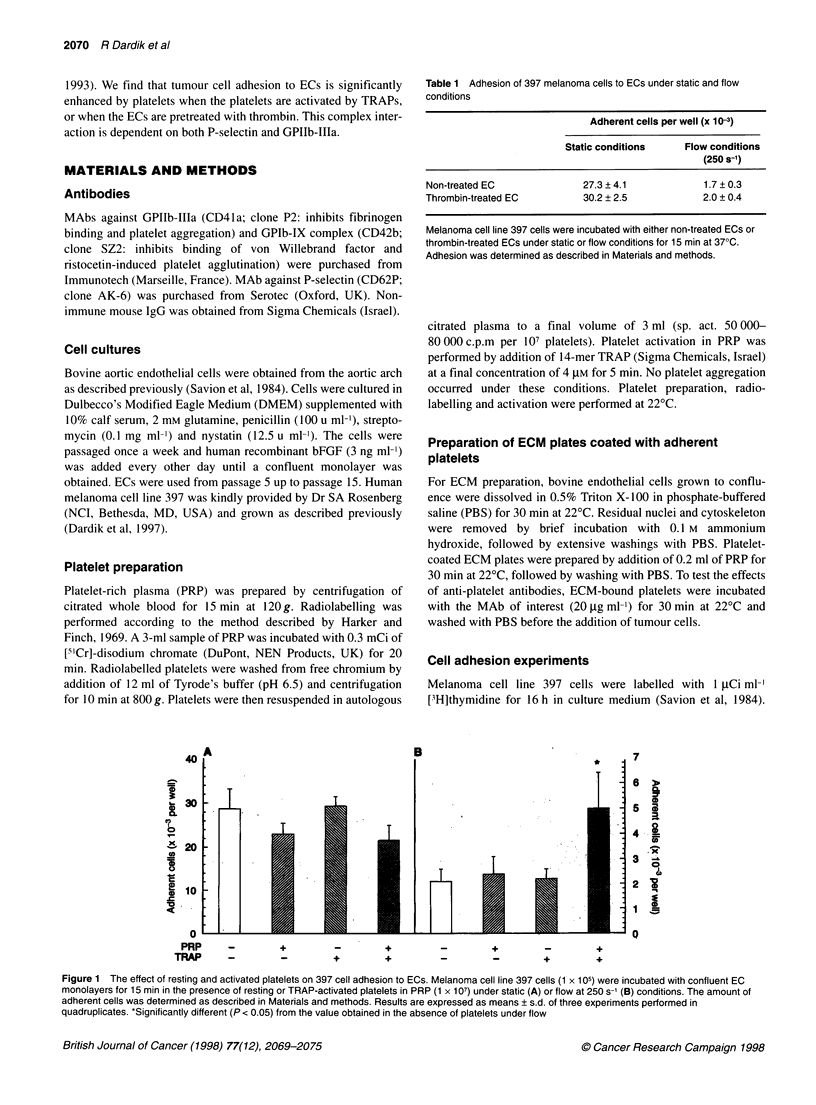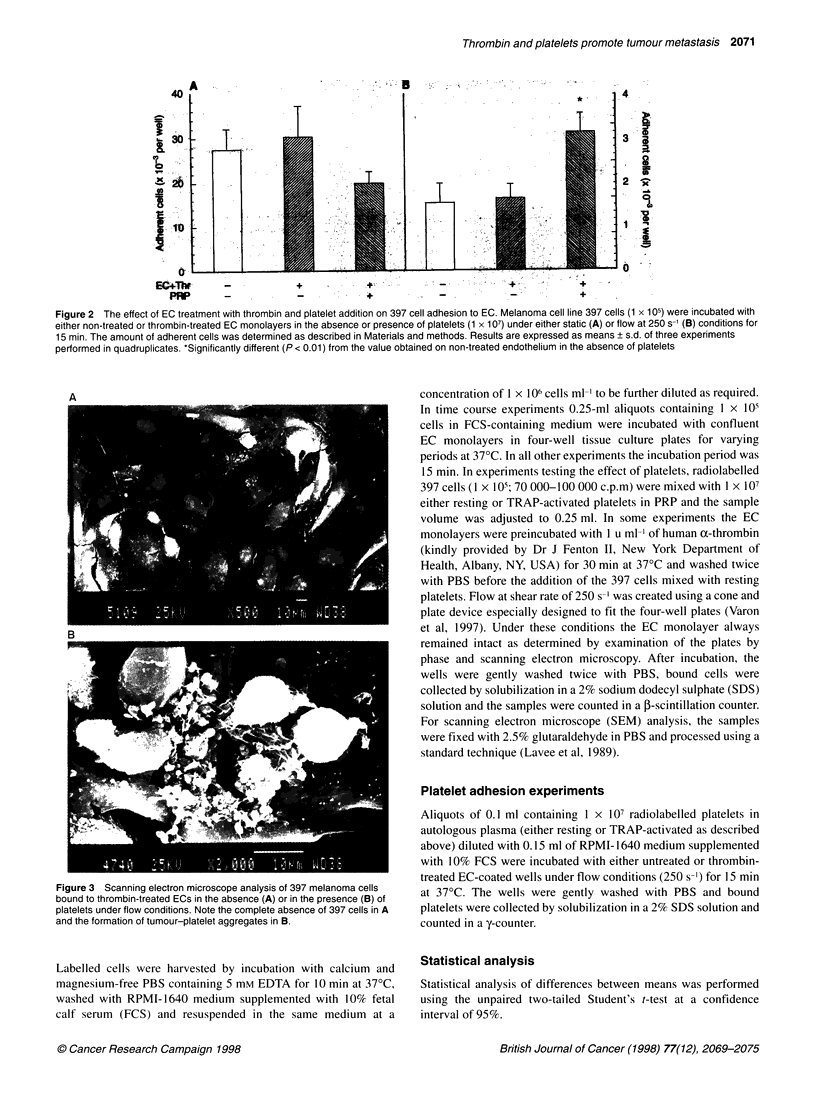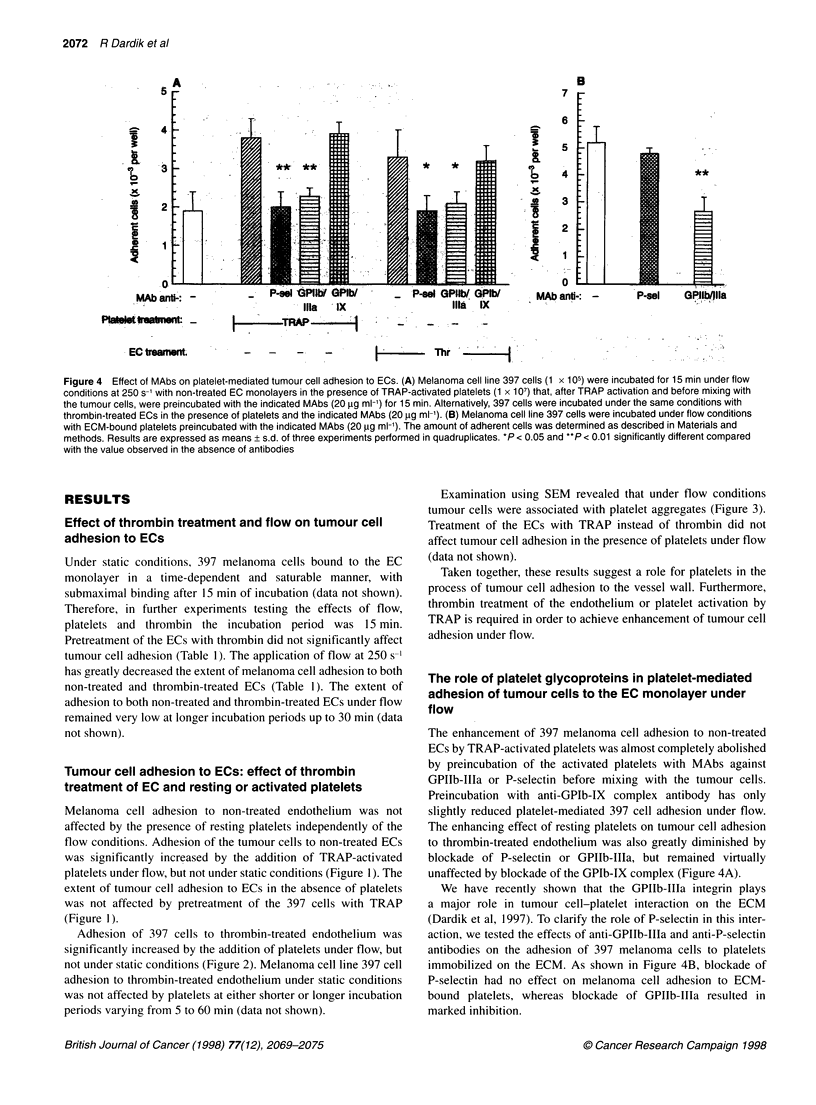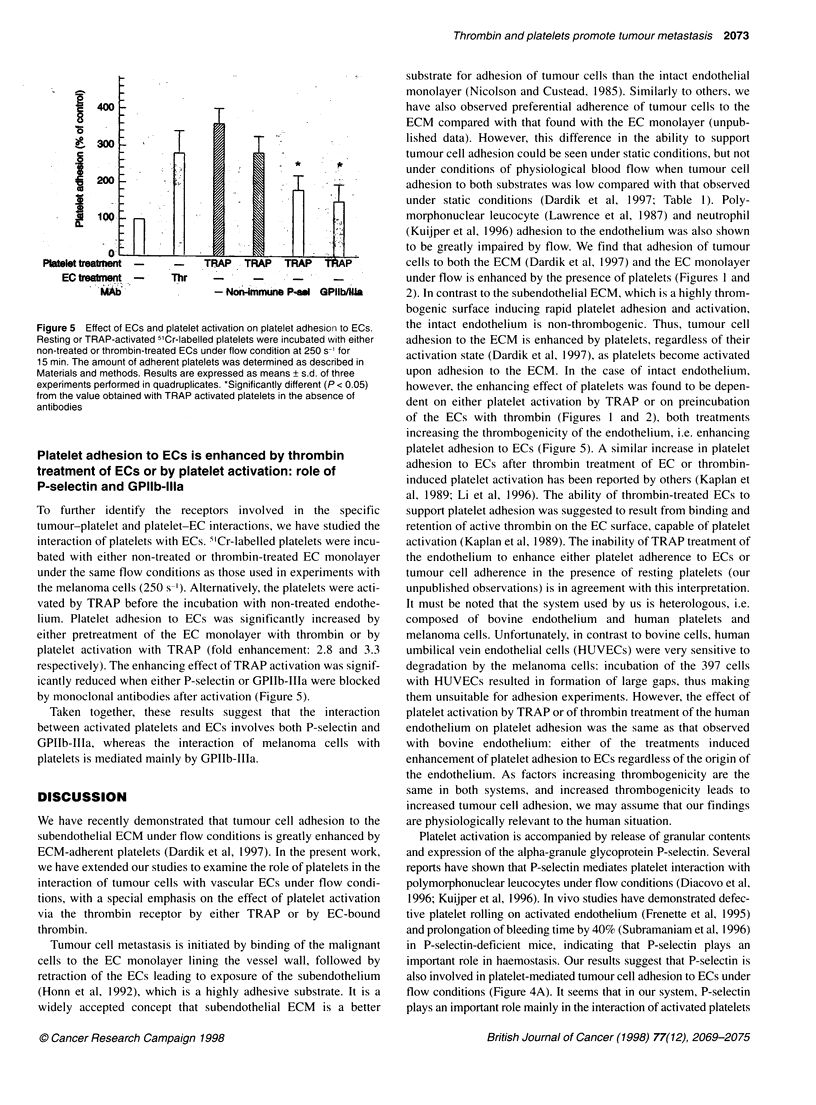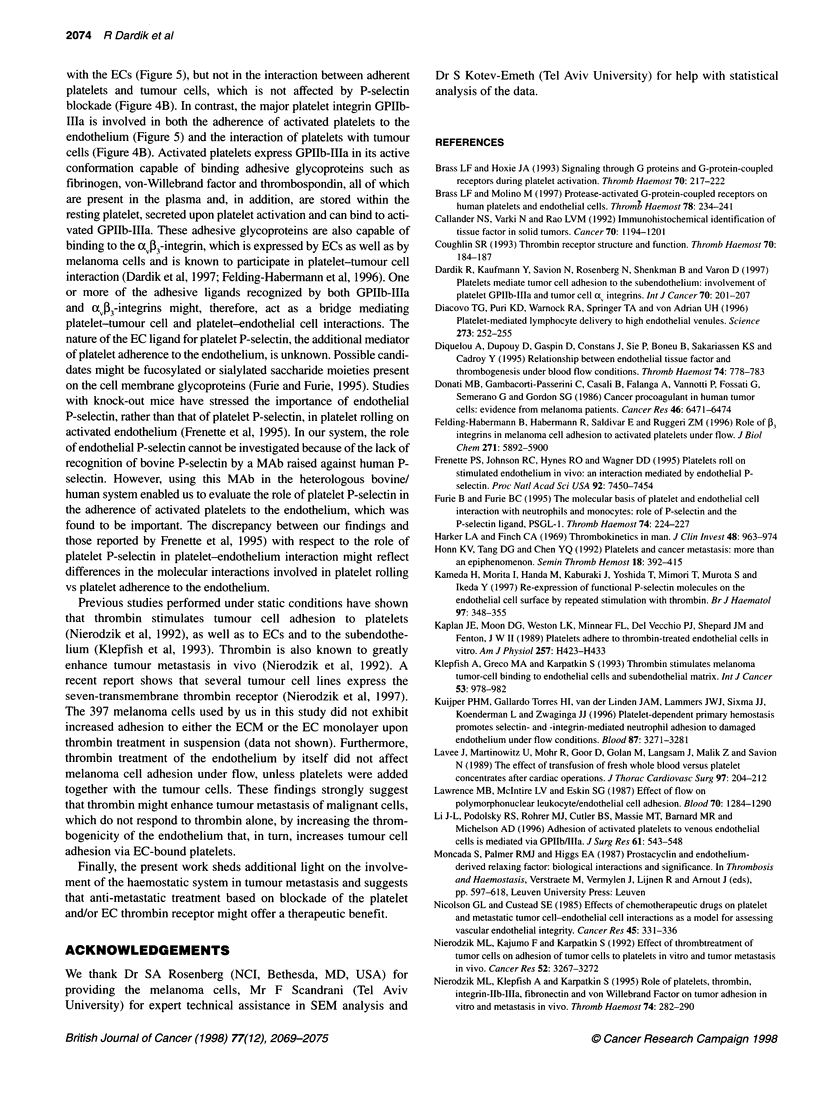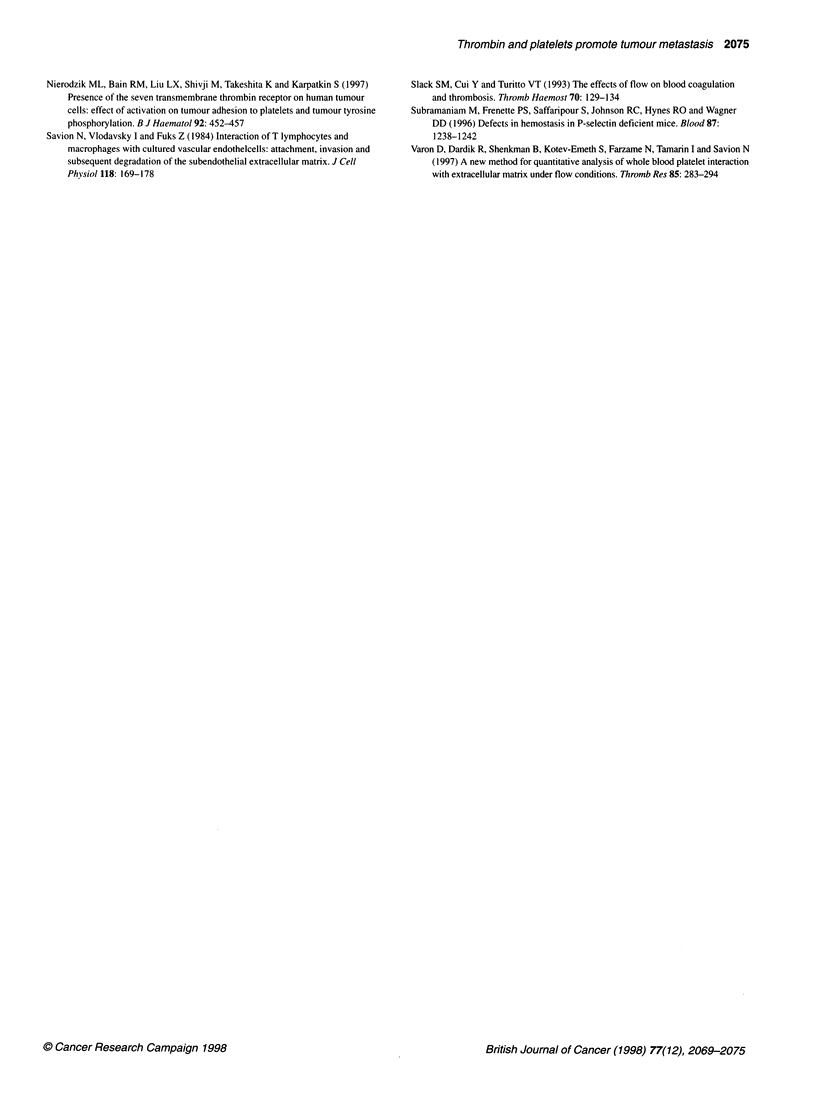# Thrombin promotes platelet-mediated melanoma cell adhesion to endothelial cells under flow conditions: role of platelet glycoproteins P-selectin and GPIIb-IIIA.

**DOI:** 10.1038/bjc.1998.349

**Published:** 1998-06

**Authors:** R. Dardik, N. Savion, Y. Kaufmann, D. Varon

**Affiliations:** National Hemophilia Center and Institute of Thrombosis and Hemostasis, Sheba Medical Center, Tel Hashomer, Israel.

## Abstract

**Images:**


*British Journal of Cancer* (1998) **77**(12), 2069–2075
© 1998 Cancer Research Campaign

### Thrombin promotes platelet-mediated melanoma cell adhesion to endothelial cells under flow conditions: role of platelet glycoproteins P-selectin and GPIIb-IIIA

**R Dardik[1], N Savion[2], Y Kaufmann[3] and D Varon[1]**

[1]National Hemophilia Center and Institute of Thrombosis and Hemostasis, Sheba Medical Center, Tel Hashomer 52621, Israel; [2]Goldschleger Eye Research Institute, Tel Aviv University, Tel Aviv, Israel; [3]Institute of Hematology, Sheba Medical Center, Tel Hashomer, Israel

**Summary** We investigated the role of platelets in human melanoma cell (line 397) interaction with vascular endothelial cells (ECs) under flow conditions. The ability of the tumour cells to adhere to the EC monolayer was significantly reduced by application of flow at a shear rate of 250 $s^{-1}$. A 2.2-fold increase in tumour cell adhesion to ECs under flow was observed upon addition of thrombin receptor agonist peptide (TRAP)-activated platelets but not resting platelets. A similar increase (2.5-fold) in tumour cell adhesion to ECs under flow was observed when the tumour cells were incubated with resting platelets on thrombin-treated ECs. However, thrombin treatment of the ECs alone had no effect on tumour cell adhesion in the absence of platelets. The enhancement of tumour cell adhesion to ECs by TRAP-activated platelets was virtually abolished by blockade of the platelet glycoproteins P-selectin and GPIIb-IIIa by monoclonal antibodies. Blockade of P-selectin also inhibited the direct adhesion of TRAP-activated platelets to ECs, but did not affect the interaction of the tumour cells with platelets immobilized on subendothelial extracellular matrix (ECM). Blockade of GPIIb-IIIa inhibited both platelet–EC and platelet–tumor cell interactions. Our results indicate that tumour cell adhesion to the endothelium under flow is enhanced by platelets under conditions that allow platelet adhesion to ECs. Inhibition studies suggest that activated platelet adhesion to ECs is mediated by P-selectin and GPIIb-IIIA, and tumour cell adhesion to EC-bound platelets – mainly by GPIIb-IIIa.

**Keywords:** thrombin receptor; platelet activation; adhesion; melanoma cell; endothelium

Tumour cell adhesion to the vessel wall is one of the earliest events initiating tumour metastasis. Numerous in vivo and in vitro studies have demonstrated that platelets greatly potentiate tumour metastasis by virtue of their ability to interact with both tumour cells and vessel wall components (for review see Honn et al, 1992 and Nierodzik et al, 1995). Intact vascular endothelium covering the vessel wall is normally non-thrombogenic. However, in vitro studies have shown that platelets can adhere to endothelial cells (ECs) stimulated with certain agonists, e.g. lipopolysaccharides or interleukin I β (Diquelou et al, 1995). It was also reported that thrombin treatment of ECs alone (Kaplan et al, 1989) or of ECs and platelets simultaneously (Li et al, 1996) leads to increased platelet adherence to the endothelium.

The thrombin receptor present on platelets and ECs is a G-protein-coupled seven transmembrane domain protein. The extracellular amino terminus of the receptor contains a thrombin cleavage site located between Arg-41 and Ser-42. Cleavage at this site exposes a new amino terminus that acts as a tethered ligand binding to another transmembrane domain to cause receptor activation (Coughlin, 1993). Synthetic peptides containing the first N-terminal 5 amino acids of the tethered ligand, SFLLR, are capable of activating the thrombin receptor without thrombin cleavage. These peptides, known as thrombin receptor agonist peptides (TRAPs), are assumed to mimic the effect of thrombin by inducing a conformational change in the receptor required to activate the G-proteins that are coupled to its cytoplasmic domain (Coughlin, 1993). Thrombin is a potent platelet agonist causing phosphoinositide hydrolysis, increase in cytosolic $Ca^{2+}$, suppression of cAMP synthesis and protein phosphorylation on serine, threonine and tyrosine residues (Brass and Hoxie, 1993). The intracellular events triggered by thrombin receptor activation on ECs are activation of phospholipases $A_2$, C and D and elevation of cytosolic $Ca^{2+}$ (Brass and Molino, 1997). In addition, thrombin stimulates secretion of platelet-activating factor, platelet-derived growth factor, von Willebrand factor (Brass and Molino, 1997), as well as secretion and surface expression of P-selectin (Kameda et al, 1997).

Normal endothelium prevents activation of the coagulation cascade and platelet stimulation by a variety of mechanisms (Moncada et al, 1987). However, many tumour cells express tissue factor (Callander, 1992) or cancer procoagulant (Donati et al, 1986), and are thus capable of activating the coagulation system leading to increased thrombin generation and, therefore, increased thrombogenicity of the endothelium. In the present study, we addressed the potential contribution of increased thrombogenicity to tumour metastasis. We have recently shown that tumour cell adhesion to the subendothelial extracellular matrix (ECM) occurring under static conditions is almost completely abolished upon introduction of physiological flow conditions and that ECM-bound platelets can partially restore tumour cell adhesion to the ECM under flow (Dardik et al, 1997). In the present work, we extended our studies and investigated the role of platelet and/or EC activation via the thrombin receptor in platelet-mediated adhesion of tumour cells to intact vascular endothelium under flow conditions resembling those existing in the arteries (Slack et al,

*Received 30 June 1997*
*Revised 23 December 1997*
*Accepted 5 January 1998*

*Correspondence to:* D Varon

1993). We find that tumour cell adhesion to ECs is significantly enhanced by platelets when the platelets are activated by TRAPs, or when the ECs are pretreated with thrombin. This complex interaction is dependent on both P-selectin and GPIIb-IIIa.

### MATERIALS AND METHODS

### Antibodies

MAbs against GPIIb-IIIa (CD41a; clone P2: inhibits fibrinogen binding and platelet aggregation) and GPIb-IX complex (CD42b; clone SZ2: inhibits binding of von Willebrand factor and ristocetin-induced platelet agglutination) were purchased from Immunotech (Marseille, France). MAb against P-selectin (CD62P; clone AK-6) was purchased from Serotec (Oxford, UK). Non-immune mouse IgG was obtained from Sigma Chemicals (Israel).

### Cell cultures

Bovine aortic endothelial cells were obtained from the aortic arch as described previously (Savion et al, 1984). Cells were cultured in Dulbecco's Modified Eagle Medium (DMEM) supplemented with 10% calf serum, 2 mM glutamine, penicillin (100 u $ml^{-1}$), streptomycin (0.1 mg $ml^{-1}$) and nystatin (12.5 u $ml^{-1}$). The cells were passaged once a week and human recombinant bFGF (3 ng $ml^{-1}$) was added every other day until a confluent monolayer was obtained. ECs were used from passage 5 up to passage 15. Human melanoma cell line 397 was kindly provided by Dr SA Rosenberg (NCI, Bethesda, MD, USA) and grown as described previously (Dardik et al, 1997).

### Platelet preparation

Platelet-rich plasma (PRP) was prepared by centrifugation of citrated whole blood for 15 min at 120 ***g***. Radiolabelling was performed according to the method described by Harker and Finch, 1969. A 3-ml sample of PRP was incubated with 0.3 mCi of [$^{51}$Cr]-disodium chromate (DuPont, NEN Products, UK) for 20 min. Radiolabelled platelets were washed from free chromium by addition of 12 ml of Tyrode's buffer (pH 6.5) and centrifugation for 10 min at 800 ***g***. Platelets were then resuspended in autologous citrated plasma to a final volume of 3 ml (sp. act. 50 000–80 000 c.p.m per $10^7$ platelets). Platelet activation in PRP was performed by addition of 14-mer TRAP (Sigma Chemicals, Israel) at a final concentration of 4 μM for 5 min. No platelet aggregation occurred under these conditions. Platelet preparation, radiolabelling and activation were performed at 22°C.

**Table 1** Adhesion of 397 melanoma cells to ECs under static and flow conditions

| | Adherent cells per well (x $10^{-3}$) | |
|---|---|---|
| | Static conditions | Flow conditions (250 $s^{-1}$) |
| Non-treated EC | 27.3 ± 4.1 | 1.7 ± 0.3 |
| Thrombin-treated EC | 30.2 ± 2.5 | 2.0 ± 0.4 |

Melanoma cell line 397 cells were incubated with either non-treated ECs or thrombin-treated ECs under static or flow conditions for 15 min at 37°C. Adhesion was determined as described in Materials and methods.

### Preparation of ECM plates coated with adherent platelets

For ECM preparation, bovine endothelial cells grown to confluence were dissolved in 0.5% Triton X-100 in phosphate-buffered saline (PBS) for 30 min at 22°C. Residual nuclei and cytoskeleton were removed by brief incubation with 0.1 M ammonium hydroxide, followed by extensive washings with PBS. Platelet-coated ECM plates were prepared by addition of 0.2 ml of PRP for 30 min at 22°C, followed by washing with PBS. To test the effects of anti-platelet antibodies, ECM-bound platelets were incubated with the MAb of interest (20 μg $ml^{-1}$) for 30 min at 22°C and washed with PBS before the addition of tumour cells.

### Cell adhesion experiments

Melanoma cell line 397 cells were labelled with 1 μCi $ml^{-1}$ [$^3$H]thymidine for 16 h in culture medium (Savion et al, 1984).

**Figure 1** The effect of resting and activated platelets on 397 cell adhesion to ECs. Melanoma cell line 397 cells (1 × $10^5$) were incubated with confluent EC monolayers for 15 min in the presence of resting or TRAP-activated platelets in PRP (1 × $10^7$) under static (**A**) or flow at 250 $s^{-1}$ (**B**) conditions. The amount of adherent cells was determined as described in Materials and methods. Results are expressed as means ± s.d. of three experiments performed in quadruplicates. *Significantly different ($P < 0.05$) from the value obtained in the absence of platelets under flow

 *© Cancer Research Campaign 1998*

**Figure 2** The effect of EC treatment with thrombin and platelet addition on 397 cell adhesion to EC. Melanoma cell line 397 cells ($1 \times 10^5$) were incubated with either non-treated or thrombin-treated EC monolayers in the absence or presence of platelets ($1 \times 10^7$) under either static (**A**) or flow at 250 $s^{-1}$ (**B**) conditions for 15 min. The amount of adherent cells was determined as described in Materials and methods. Results are expressed as means ± s.d. of three experiments performed in quadruplicates. *Significantly different ($P < 0.01$) from the value obtained on non-treated endothelium in the absence of platelets

**Figure 3** Scanning electron microscope analysis of 397 melanoma cells bound to thrombin-treated ECs in the absence (**A**) or in the presence (**B**) of platelets under flow conditions. Note the complete absence of 397 cells in **A** and the formation of tumour–platelet aggregates in **B**.

Labelled cells were harvested by incubation with calcium and magnesium-free PBS containing 5 mM EDTA for 10 min at 37°C, washed with RPMI-1640 medium supplemented with 10% fetal calf serum (FCS) and resuspended in the same medium at a concentration of $1 \times 10^6$ cells $ml^{-1}$ to be further diluted as required. In time course experiments 0.25-ml aliquots containing $1 \times 10^5$ cells in FCS-containing medium were incubated with confluent EC monolayers in four-well tissue culture plates for varying periods at 37°C. In all other experiments the incubation period was 15 min. In experiments testing the effect of platelets, radiolabelled 397 cells ($1 \times 10^5$; 70 000–100 000 c.p.m) were mixed with $1 \times 10^7$ either resting or TRAP-activated platelets in PRP and the sample volume was adjusted to 0.25 ml. In some experiments the EC monolayers were preincubated with 1 u $ml^{-1}$ of human α-thrombin (kindly provided by Dr J Fenton II, New York Department of Health, Albany, NY, USA) for 30 min at 37°C and washed twice with PBS before the addition of the 397 cells mixed with resting platelets. Flow at shear rate of 250 $s^{-1}$ was created using a cone and plate device especially designed to fit the four-well plates (Varon et al, 1997). Under these conditions the EC monolayer always remained intact as determined by examination of the plates by phase and scanning electron microscopy. After incubation, the wells were gently washed twice with PBS, bound cells were collected by solubilization in a 2% sodium dodecyl sulphate (SDS) solution and the samples were counted in a β-scintillation counter. For scanning electron microscope (SEM) analysis, the samples were fixed with 2.5% glutaraldehyde in PBS and processed using a standard technique (Lavee et al, 1989).

### Platelet adhesion experiments

Aliquots of 0.1 ml containing $1 \times 10^7$ radiolabelled platelets in autologous plasma (either resting or TRAP-activated as described above) diluted with 0.15 ml of RPMI-1640 medium supplemented with 10% FCS were incubated with either untreated or thrombin-treated EC-coated wells under flow conditions (250 $s^{-1}$) for 15 min at 37°C. The wells were gently washed with PBS and bound platelets were collected by solubilization in a 2% SDS solution and counted in a γ-counter.

### Statistical analysis

Statistical analysis of differences between means was performed using the unpaired two-tailed Student's *t*-test at a confidence interval of 95%.

© Cancer Research Campaign 1998

**Figure 4** Effect of MAbs on platelet-mediated tumour cell adhesion to ECs. (**A**) Melanoma cell line 397 cells (1 × 10$^5$) were incubated for 15 min under flow conditions at 250 s$^{-1}$ with non-treated EC monolayers in the presence of TRAP-activated platelets (1 × 10$^7$) that, after TRAP activation and before mixing with the tumour cells, were preincubated with the indicated MAbs (20 μg ml$^{-1}$) for 15 min. Alternatively, 397 cells were incubated under the same conditions with thrombin-treated ECs in the presence of platelets and the indicated MAbs (20 μg ml$^{-1}$). (**B**) Melanoma cell line 397 cells were incubated under flow conditions with ECM-bound platelets preincubated with the indicated MAbs (20 μg ml$^{-1}$). The amount of adherent cells was determined as described in Materials and methods. Results are expressed as means ± s.d. of three experiments performed in quadruplicates. *$P$ < 0.05 and **$P$ < 0.01 significantly different compared with the value observed in the absence of antibodies

### RESULTS

### Effect of thrombin treatment and flow on tumour cell adhesion to ECs

Under static conditions, 397 melanoma cells bound to the EC monolayer in a time-dependent and saturable manner, with submaximal binding after 15 min of incubation (data not shown). Therefore, in further experiments testing the effects of flow, platelets and thrombin the incubation period was 15 min. Pretreatment of the ECs with thrombin did not significantly affect tumour cell adhesion (Table 1). The application of flow at 250 s$^{-1}$ has greatly decreased the extent of melanoma cell adhesion to both non-treated and thrombin-treated ECs (Table 1). The extent of adhesion to both non-treated and thrombin-treated ECs under flow remained very low at longer incubation periods up to 30 min (data not shown).

### Tumour cell adhesion to ECs: effect of thrombin treatment of EC and resting or activated platelets

Melanoma cell adhesion to non-treated endothelium was not affected by the presence of resting platelets independently of the flow conditions. Adhesion of the tumour cells to non-treated ECs was significantly increased by the addition of TRAP-activated platelets under flow, but not under static conditions (Figure 1). The extent of tumour cell adhesion to ECs in the absence of platelets was not affected by pretreatment of the 397 cells with TRAP (Figure 1).

Adhesion of 397 cells to thrombin-treated endothelium was significantly increased by the addition of platelets under flow, but not under static conditions (Figure 2). Melanoma cell line 397 cell adhesion to thrombin-treated endothelium under static conditions was not affected by platelets at either shorter or longer incubation periods varying from 5 to 60 min (data not shown).

Examination using SEM revealed that under flow conditions tumour cells were associated with platelet aggregates (Figure 3). Treatment of the ECs with TRAP instead of thrombin did not affect tumour cell adhesion in the presence of platelets under flow (data not shown).

Taken together, these results suggest a role for platelets in the process of tumour cell adhesion to the vessel wall. Furthermore, thrombin treatment of the endothelium or platelet activation by TRAP is required in order to achieve enhancement of tumour cell adhesion under flow.

### The role of platelet glycoproteins in platelet-mediated adhesion of tumour cells to the EC monolayer under flow

The enhancement of 397 melanoma cell adhesion to non-treated ECs by TRAP-activated platelets was almost completely abolished by preincubation of the activated platelets with MAbs against GPIIb-IIIa or P-selectin before mixing with the tumour cells. Preincubation with anti-GPIb-IX complex antibody has only slightly reduced platelet-mediated 397 cell adhesion under flow. The enhancing effect of resting platelets on tumour cell adhesion to thrombin-treated endothelium was also greatly diminished by blockade of P-selectin or GPIIb-IIIa, but remained virtually unaffected by blockade of the GPIb-IX complex (Figure 4A).

We have recently shown that the GPIIb-IIIa integrin plays a major role in tumour cell–platelet interaction on the ECM (Dardik et al, 1997). To clarify the role of P-selectin in this interaction, we tested the effects of anti-GPIIb-IIIa and anti-P-selectin antibodies on the adhesion of 397 melanoma cells to platelets immobilized on the ECM. As shown in Figure 4B, blockade of P-selectin had no effect on melanoma cell adhesion to ECM-bound platelets, whereas blockade of GPIIb-IIIa resulted in marked inhibition.

 © Cancer Research Campaign 1998

**Figure 5** Effect of ECs and platelet activation on platelet adhesion to ECs. Resting or TRAP-activated $^{51}$Cr-labelled platelets were incubated with either non-treated or thrombin-treated ECs under flow condition at 250 $s^{-1}$ for 15 min. The amount of adherent platelets was determined as described in Materials and methods. Results are expressed as means ± s.d. of three experiments performed in quadruplicates. *Significantly different ($P < 0.05$) from the value obtained with TRAP activated platelets in the absence of antibodies

### Platelet adhesion to ECs is enhanced by thrombin treatment of ECs or by platelet activation: role of P-selectin and GPIIb-IIIa

To further identify the receptors involved in the specific tumour–platelet and platelet–EC interactions, we have studied the interaction of platelets with ECs. $^{51}$Cr-labelled platelets were incubated with either non-treated or thrombin-treated EC monolayer under the same flow conditions as those used in experiments with the melanoma cells (250 $s^{-1}$). Alternatively, the platelets were activated by TRAP before the incubation with non-treated endothelium. Platelet adhesion to ECs was significantly increased by either pretreatment of the EC monolayer with thrombin or by platelet activation with TRAP (fold enhancement: 2.8 and 3.3 respectively). The enhancing effect of TRAP activation was significantly reduced when either P-selectin or GPIIb-IIIa were blocked by monoclonal antibodies after activation (Figure 5).

Taken together, these results suggest that the interaction between activated platelets and ECs involves both P-selectin and GPIIb-IIIa, whereas the interaction of melanoma cells with platelets is mediated mainly by GPIIb-IIIa.

### DISCUSSION

We have recently demonstrated that tumour cell adhesion to the subendothelial ECM under flow conditions is greatly enhanced by ECM-adherent platelets (Dardik et al, 1997). In the present work, we have extended our studies to examine the role of platelets in the interaction of tumour cells with vascular ECs under flow conditions, with a special emphasis on the effect of platelet activation via the thrombin receptor by either TRAP or by EC-bound thrombin.

Tumour cell metastasis is initiated by binding of the malignant cells to the EC monolayer lining the vessel wall, followed by retraction of the ECs leading to exposure of the subendothelium (Honn et al, 1992), which is a highly adhesive substrate. It is a widely accepted concept that subendothelial ECM is a better substrate for adhesion of tumour cells than the intact endothelial monolayer (Nicolson and Custead, 1985). Similarly to others, we have also observed preferential adherence of tumour cells to the ECM compared with that found with the EC monolayer (unpublished data). However, this difference in the ability to support tumour cell adhesion could be seen under static conditions, but not under conditions of physiological blood flow when tumour cell adhesion to both substrates was low compared with that observed under static conditions (Dardik et al, 1997; Table 1). Polymorphonuclear leucocyte (Lawrence et al, 1987) and neutrophil (Kuijper et al, 1996) adhesion to the endothelium was also shown to be greatly impaired by flow. We find that adhesion of tumour cells to both the ECM (Dardik et al, 1997) and the EC monolayer under flow is enhanced by the presence of platelets (Figures 1 and 2). In contrast to the subendothelial ECM, which is a highly thrombogenic surface inducing rapid platelet adhesion and activation, the intact endothelium is non-thrombogenic. Thus, tumour cell adhesion to the ECM is enhanced by platelets, regardless of their activation state (Dardik et al, 1997), as platelets become activated upon adhesion to the ECM. In the case of intact endothelium, however, the enhancing effect of platelets was found to be dependent on either platelet activation by TRAP or on preincubation of the ECs with thrombin (Figures 1 and 2), both treatments increasing the thrombogenicity of the endothelium, i.e. enhancing platelet adhesion to ECs (Figure 5). A similar increase in platelet adhesion to ECs after thrombin treatment of EC or thrombin-induced platelet activation has been reported by others (Kaplan et al, 1989; Li et al, 1996). The ability of thrombin-treated ECs to support platelet adhesion was suggested to result from binding and retention of active thrombin on the EC surface, capable of platelet activation (Kaplan et al, 1989). The inability of TRAP treatment of the endothelium to enhance either platelet adherence to ECs or tumour cell adherence in the presence of resting platelets (our unpublished observations) is in agreement with this interpretation. It must be noted that the system used by us is heterologous, i.e. composed of bovine endothelium and human platelets and melanoma cells. Unfortunately, in contrast to bovine cells, human umbilical vein endothelial cells (HUVECs) were very sensitive to degradation by the melanoma cells: incubation of the 397 cells with HUVECs resulted in formation of large gaps, thus making them unsuitable for adhesion experiments. However, the effect of platelet activation by TRAP or of thrombin treatment of the human endothelium on platelet adhesion was the same as that observed with bovine endothelium: either of the treatments induced enhancement of platelet adhesion to ECs regardless of the origin of the endothelium. As factors increasing thrombogenicity are the same in both systems, and increased thrombogenicity leads to increased tumour cell adhesion, we may assume that our findings are physiologically relevant to the human situation.

Platelet activation is accompanied by release of granular contents and expression of the alpha-granule glycoprotein P-selectin. Several reports have shown that P-selectin mediates platelet interaction with polymorphonuclear leucocytes under flow conditions (Diacovo et al, 1996; Kuijper et al, 1996). In vivo studies have demonstrated defective platelet rolling on activated endothelium (Frenette et al, 1995) and prolongation of bleeding time by 40% (Subramaniam et al, 1996) in P-selectin-deficient mice, indicating that P-selectin plays an important role in haemostasis. Our results suggest that P-selectin is also involved in platelet-mediated tumour cell adhesion to ECs under flow conditions (Figure 4A). It seems that in our system, P-selectin plays an important role mainly in the interaction of activated platelets

© Cancer Research Campaign 1998

with the ECs (Figure 5), but not in the interaction between adherent platelets and tumour cells, which is not affected by P-selectin blockade (Figure 4B). In contrast, the major platelet integrin GPIIb-IIIa is involved in both the adherence of activated platelets to the endothelium (Figure 5) and the interaction of platelets with tumour cells (Figure 4B). Activated platelets express GPIIb-IIIa in its active conformation capable of binding adhesive glycoproteins such as fibrinogen, von-Willebrand factor and thrombospondin, all of which are present in the plasma and, in addition, are stored within the resting platelet, secreted upon platelet activation and can bind to activated GPIIb-IIIa. These adhesive glycoproteins are also capable of binding to the $\alpha_v\beta_3$-integrin, which is expressed by ECs as well as by melanoma cells and is known to participate in platelet–tumour cell interaction (Dardik et al, 1997; Felding-Habermann et al, 1996). One or more of the adhesive ligands recognized by both GPIIb-IIIa and $\alpha_v\beta_3$-integrins might, therefore, act as a bridge mediating platelet–tumour cell and platelet–endothelial cell interactions. The nature of the EC ligand for platelet P-selectin, the additional mediator of platelet adherence to the endothelium, is unknown. Possible candidates might be fucosylated or sialylated saccharide moieties present on the cell membrane glycoproteins (Furie and Furie, 1995). Studies with knock-out mice have stressed the importance of endothelial P-selectin, rather than that of platelet P-selectin, in platelet rolling on activated endothelium (Frenette et al, 1995). In our system, the role of endothelial P-selectin cannot be investigated because of the lack of recognition of bovine P-selectin by a MAb raised against human P-selectin. However, using this MAb in the heterologous bovine/human system enabled us to evaluate the role of platelet P-selectin in the adherence of activated platelets to the endothelium, which was found to be important. The discrepancy between our findings and those reported by Frenette et al, 1995) with respect to the role of platelet P-selectin in platelet–endothelium interaction might reflect differences in the molecular interactions involved in platelet rolling vs platelet adherence to the endothelium.

Previous studies performed under static conditions have shown that thrombin stimulates tumour cell adhesion to platelets (Nierodzik et al, 1992), as well as to ECs and to the subendothelium (Klepfish et al, 1993). Thrombin is also known to greatly enhance tumour metastasis in vivo (Nierodzik et al, 1992). A recent report shows that several tumour cell lines express the seven-transmembrane thrombin receptor (Nierodzik et al, 1997). The 397 melanoma cells used by us in this study did not exhibit increased adhesion to either the ECM or the EC monolayer upon thrombin treatment in suspension (data not shown). Furthermore, thrombin treatment of the endothelium by itself did not affect melanoma cell adhesion under flow, unless platelets were added together with the tumour cells. These findings strongly suggest that thrombin might enhance tumour metastasis of malignant cells, which do not respond to thrombin alone, by increasing the thrombogenicity of the endothelium that, in turn, increases tumour cell adhesion via EC-bound platelets.

Finally, the present work sheds additional light on the involvement of the haemostatic system in tumour metastasis and suggests that anti-metastatic treatment based on blockade of the platelet and/or EC thrombin receptor might offer a therapeutic benefit.

### ACKNOWLEDGEMENTS

We thank Dr SA Rosenberg (NCI, Bethesda, MD, USA) for providing the melanoma cells, Mr F Scandrani (Tel Aviv University) for expert technical assistance in SEM analysis and Dr S Kotev-Emeth (Tel Aviv University) for help with statistical analysis of the data.

### REFERENCES

Brass LF and Hoxie JA (1993) Signaling through G proteins and G-protein-coupled receptors during platelet activation. *Thromb Haemost* **70**: 217–222

Brass LF and Molino M (1997) Protease-activated G-protein-coupled receptors on human platelets and endothelial cells. *Thromb Haemost* **78**: 234–241

Callander NS, Varki N and Rao LVM (1992) Immunohistochemical identification of tissue factor in solid tumors. *Cancer* **70**: 1194–1201

Coughlin SR (1993) Thrombin receptor structure and function. *Thromb Haemost* **70**: 184–187

Dardik R, Kaufmann Y, Savion N, Rosenberg N, Shenkman B and Varon D (1997) Platelets mediate tumor cell adhesion to the subendothelium: involvement of platelet GPIIb-IIIa and tumor cell $\alpha_v$ integrins. *Int J Cancer* **70**: 201–207

Diacovo TG, Puri KD, Warnock RA, Springer TA and von Adrian UH (1996) Platelet-mediated lymphocyte delivery to high endothelial venules. *Science* **273**: 252–255

Diquelou A, Dupouy D, Gaspin D, Constans J, Sie P, Boneu B, Sakariassen KS and Cadroy Y (1995) Relationship between endothelial tissue factor and thrombogenesis under blood flow conditions. *Thromb Haemost* **74**: 778–783

Donati MB, Gambacorti-Passerini C, Casali B, Falanga A, Vannotti P, Fossati G, Semerano G and Gordon SG (1986) Cancer procoagulant in human tumor cells: evidence from melanoma patients. *Cancer Res* **46**: 6471–6474

Felding-Habermann B, Habermann R, Saldivar E and Ruggeri ZM (1996) Role of $\beta_3$ integrins in melanoma cell adhesion to activated platelets under flow. *J Biol Chem* **271**: 5892–5900

Frenette PS, Johnson RC, Hynes RO and Wagner DD (1995) Platelets roll on stimulated endothelium in vivo: an interaction mediated by endothelial P-selectin. *Proc Natl Acad Sci USA* **92**: 7450–7454

Furie B and Furie BC (1995) The molecular basis of platelet and endothelial cell interaction with neutrophils and monocytes: role of P-selectin and the P-selectin ligand, PSGL-1. *Thromb Haemost* **74**: 224–227

Harker LA and Finch CA (1969) Thrombokinetics in man. *J Clin Invest* **48**: 963–974

Honn KV, Tang DG and Chen YQ (1992) Platelets and cancer metastasis: more than an epiphenomenon. *Semin Thromb Hemost* **18**: 392–415

Kameda H, Morita I, Handa M, Kaburaki J, Yoshida T, Mimori T, Murota S and Ikeda Y (1997) Re-expression of functional P-selectin molecules on the endothelial cell surface by repeated stimulation with thrombin. *Br J Haematol* **97**: 348–355

Kaplan JE, Moon DG, Weston LK, Minnear FL, Del Vecchio PJ, Shepard JM and Fenton, J W II (1989) Platelets adhere to thrombin-treated endothelial cells in vitro. *Am J Physiol* **257**: H423–H433

Klepfish A, Greco MA and Karpatkin S (1993) Thrombin stimulates melanoma tumor-cell binding to endothelial cells and subendothelial matrix. *Int J Cancer* **53**: 978–982

Kuijper PHM, Gallardo Torres HI, van der Linden JAM, Lammers JWJ, Sixma JJ, Koenderman L and Zwaginga JJ (1996) Platelet-dependent primary hemostasis promotes selectin- and -integrin-mediated neutrophil adhesion to damaged endothelium under flow conditions. *Blood* **87**: 3271–3281

Lavee J, Martinowitz U, Mohr R, Goor D, Golan M, Langsam J, Malik Z and Savion N (1989) The effect of transfusion of fresh whole blood versus platelet concentrates after cardiac operations. *J Thorac Cardiovasc Surg* **97**: 204–212

Lawrence MB, McIntire LV and Eskin SG (1987) Effect of flow on polymorphonuclear leukocyte/endothelial cell adhesion. *Blood* **70**: 1284–1290

Li J-L, Podolsky RS, Rohrer MJ, Cutler BS, Massie MT, Barnard MR and Michelson AD (1996) Adhesion of activated platelets to venous endothelial cells is mediated via GPIIb/IIIa. *J Surg Res* **61**: 543–548

Moncada S, Palmer RMJ and Higgs EA (1987) Prostacyclin and endothelium-derived relaxing factor: biological interactions and significance. In *Thrombosis and Haemostasis*, Verstraete M, Vermylen J, Lijnen R and Arnout J (eds), pp. 597–618, Leuven University Press: Leuven

Nicolson GL and Custead SE (1985) Effects of chemotherapeutic drugs on platelet and metastatic tumor cell–endothelial cell interactions as a model for assessing vascular endothelial integrity. *Cancer Res* **45**: 331–336

Nierodzik ML, Kajumo F and Karpatkin S (1992) Effect of thrombtreatment of tumor cells on adhesion of tumor cells to platelets in vitro and tumor metastasis in vivo. *Cancer Res* **52**: 3267–3272

Nierodzik ML, Klepfish A and Karpatkin S (1995) Role of platelets, thrombin, integrin-IIb-IIIa, fibronectin and von Willebrand Factor on tumor adhesion in vitro and metastasis in vivo. *Thromb Haemost* **74**: 282–290

 © Cancer Research Campaign 1998

Nierodzik ML, Bain RM, Liu LX, Shivji M, Takeshita K and Karpatkin S (1997) Presence of the seven transmembrane thrombin receptor on human tumour cells: effect of activation on tumour adhesion to platelets and tumour tyrosine phosphorylation. *B J Haematol* **92**: 452–457

Savion N, Vlodavsky I and Fuks Z (1984) Interaction of T lymphocytes and macrophages with cultured vascular endothelcells: attachment, invasion and subsequent degradation of the subendothelial extracellular matrix. *J Cell Physiol* **118**: 169–178

Slack SM, Cui Y and Turitto VT (1993) The effects of flow on blood coagulation and thrombosis. *Thromb Haemost* **70**: 129–134

Subramaniam M, Frenette PS, Saffaripour S, Johnson RC, Hynes RO and Wagner DD (1996) Defects in hemostasis in P-selectin deficient mice. *Blood* **87**: 1238–1242

Varon D, Dardik R, Shenkman B, Kotev-Emeth S, Farzame N, Tamarin I and Savion N (1997) A new method for quantitative analysis of whole blood platelet interaction with extracellular matrix under flow conditions. *Thromb Res* **85**: 283–294

*© Cancer Research Campaign 1998*